# Current status and issues regarding surgical education in the region: a questionnaire survey in Oita prefecture in Japan

**DOI:** 10.1186/s12909-024-05450-x

**Published:** 2024-04-24

**Authors:** Yoshitake Ueda, Takahide Kawasaki, Masafumi Inomata, Norio Shiraishi

**Affiliations:** 1 Department of Comprehensive Surgery for Community Medicine, Faculty of Medicine, Oita University, 1-1, Idaigaoka, Hasama-Machi, Yufu, Oita, 879-5593 Japan; 2 Department of Gastroenterological and Pediatric Surgery, Faculty of Medicine, Oita University, Oita, Japan

**Keywords:** Surgical education, Questionnaire survey, Regional surgical care

## Abstract

**Background:**

The shortage and aging of surgeons in regional surgical care has been remarkable, and the importance of surgical education for young surgeons in the region is only increasing. However, there are very few reports about regional surgical education. This study aimed to clarify the current status and issues regarding surgical education in regional surgical care and to examine the ideal way to provide surgical education in the region.

**Methods:**

Two questionnaire surveys were carried out. (1) “Survey on the awareness regarding the education of young surgeons” was conducted by mail in institutions where surgeons worked. (2) “Survey on the current status of surgical education for young surgeons” was conducted via the Internet with surgeons under 40 years old and mentors at the same facility.

**Results:**

There were 175 respondents to survey (1), among whom 131 (75%) surgeons were interested in educating young surgeons, and 112 (64%) were actively participating in this educating. Regarding the best evaluation methods for mentors who are educating young surgeons, the most frequent answer was “I don’t know (51%)”. The number of respondents in survey (2) was 87, including 27 (31%) young surgeons and 60 (69%) mentors. Although there was no difference between young surgeons and mentors in the level of satisfaction with the current status of young surgeons, 37% of young surgeons in urban areas were dissatisfied with their current status, compared to 0% in the regional area (*p* < 0.05).

**Conclusions:**

Although surgeons did not have confidence in their own education, the level of satisfaction among young surgeons was high even in those providing regional surgical care. Development of an evaluation system for surgical education is necessary for young surgeons to receive more effective surgical education in the region.

## Introduction

In Japan, the problem of the surgeon shortage remains unresolved. According to the Statistics of Physicians, Dentists and Pharmacists published by the Ministry of Health, Labour and Welfare in 2020, the total number of working surgeons has decreased by more than 25% over the past 20 years. As well, surgeons in Japan are becoming older. Surgeons in their 30 s have declined by approximately 30% in the past two decades with surgeons over their 40 s reaching about 70% of the total number [1]. Especially, the shortage and aging of surgeons in regional surgical care has been remarkable. Ueo et al. reported that the shortage of surgeons has become a problem in 96% of the prefectures in Japan [2]. In addition, if an upper limit on overtime work is applied as a result of the introduction of working-style reforms for physicians in the near future, the inevitable negative impact on regional surgical care will become a concern.

Meanwhile, in the surgical specialty training program for Board-Certified Surgeon in Japan, the therapeutic experiences in the region are indispensable for the acquisition of this certification. In the ever-present difficult environment surrounding surgeons, the importance of surgical education for young surgeons in the region is only increasing. However, there are very few reports examining what surgeons who provide regional surgical care think about surgical education, and the satisfaction with and requests of young surgeons in regard to the current status of surgical education.

Thus, the aim of this study was to clarify the current status and issues regarding surgical education in regional surgical care and to consider the ideal way to provide surgical education in the region.

## Methods

The two questionnaire surveys listed below were carried out in cooperation with two organizations, a surgery-related department of our university and a regional surgical care council of our prefecture.

### Questionnaire survey (1): survey on the awareness regarding the education of young surgeons

- (1)-1. Participants s of the survey

In total, 235 surgeons working in our university hospital and 33 institutions belonging to the prefectural surgical association were enrolled in this study.

- (1)-2. Aim and methods of survey

The aim of this questionnaire survey was to clarify the self-awareness as an educator of surgeons. This survey was conducted for one month in 2014 anonymously by mail to institutions where the surgeons worked. The items addressed by the questionnaire were “Background of surgeons”, “Educational training for young surgeons”, and “Evaluation methods for educational training”. The response forms were collected by mail from each institution.

### Questionnaire survey (2): survey on the current status of surgical education for young surgeons

- (2)-1. Participants of the survey

In total, 94 surgeons working in our university hospital and 16 institutions belonging to the prefectural surgical association with surgeons under 40 years old were enrolled in this study.

- (2)-2. Aim and methods of survey

The aim of this questionnaire survey was to clarify the current status of surgical education for young surgeons. This survey was conducted using the Internet for one month in 2021. The items covered in the questionnaire were “The level of satisfaction with the current status of young surgeons” and “Mutual expectations between young surgeons and their mentors”.

### Ethics approval and consent to participate

All participants of the study were provided informed consent on paper before initiation of the data collection, and their anonymity preserved. This study was approved by the Ethical Committee of Oita University Faculty of Medicine (Approval No. 2075). All methods were carried out in accordance with relevant guidelines and regulations.

### Statistical analysis

Both of these questionnaire surveys were analyzed based on the number of valid responses to the total number of responses. Analysis was performed by Mann–Whitney U test, and a *p*-value of < 0.05 was considered to indicate statistical significance. For questions that could be quantified and compared, the Likert Scale analysis method was used.

## Results

### Questionnaire survey (1): survey on the awareness of surgical education for young surgeons

- (1)-1. Response rate

The total number of valid respondents was 175, giving a response rate of 74%; 38 (22%) were surgeons working in three departments of surgery at our university hospital, and 137 (78%) were surgeons working in 33 institutions affiliated with the prefectural surgical association.

- (1)-2. Background of respondents

The mean age of the respondents was 45 years. Age groups were as follows: 11 (6%) in their 20 s, 53 (30%) in their 30 s, 54 (31%) in their 40 s, 42 (24%) in their 50 s, and 15 (9%) in their 60 s or older. There were 72 (41%) clinical instructors and 22 (13%) educators of clinical residents.

- (1)-3. Educational training for young surgeons

**Fig. 1:**
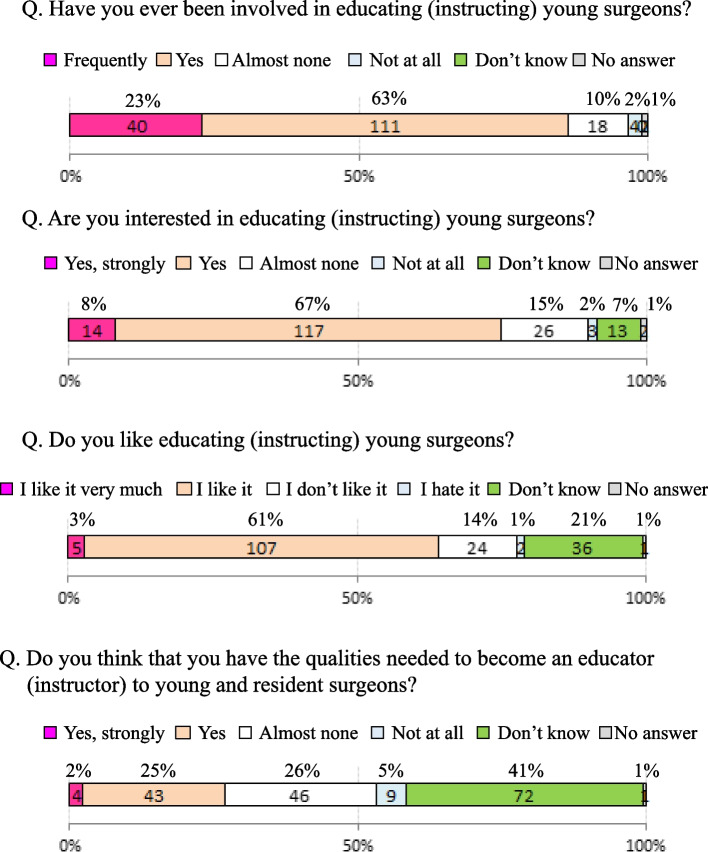
Awareness regarding the education of young surgeons

**Fig. 2:**
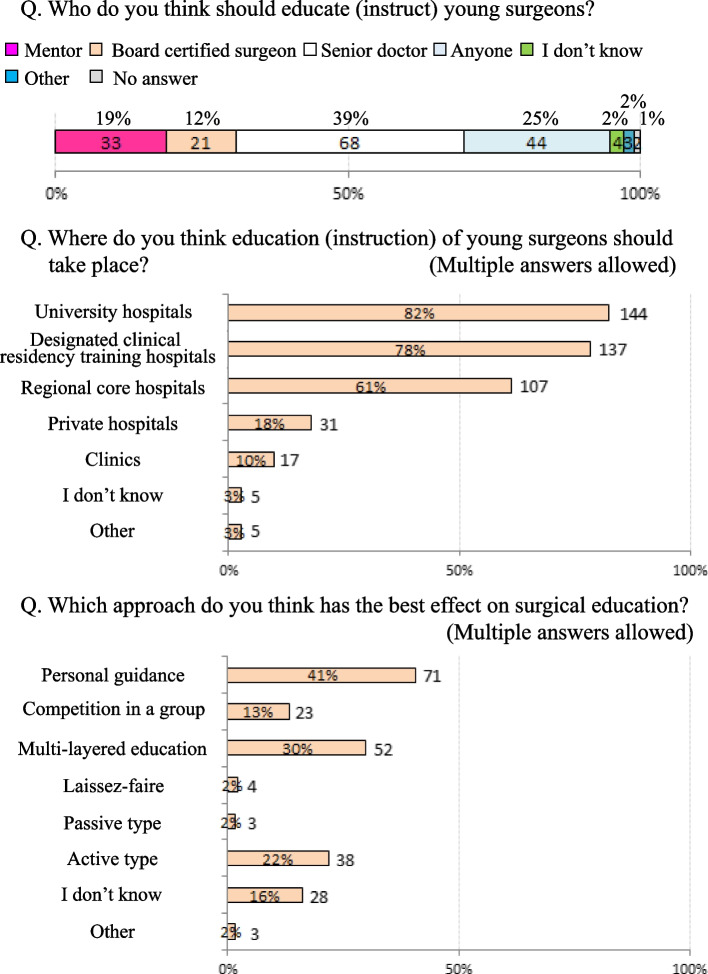
Attitude regarding the education of young surgeons

Questions that allowed multiple answers were described as multiple answers. Regarding the educational training for young surgeons, 151 (86%) surgeons had participated in the educational training of young surgeons, among whom 131 (75%) were interested in educating young surgeons, and 112 (64%) were actively participating in this education. In terms of the question of whether the responding surgeon had the qualifications to be an educator (instructor), 47 (27%) responded “yes” and 55 (31%) responded “no” (Fig. 1). For the question about who should provide educational training for young surgeons (multiple answers), “senior doctors (39%)” was the most common response, followed by “anyone (25%)” and “mentors (19%)”. Responses such as “university hospitals (82%), “designated clinical residency training hospitals (78%)” and “regional core hospitals (61%)” were given to the question of which facilities should provide educational training for young surgeons (multiple answers). Regarding the most effective educational methods (multiple answers), “personal guidance (41%)” was the most common response, followed by a “multi-layered education (30%)” and “active type (22%)” (Fig. 2).

**Fig. 3:**
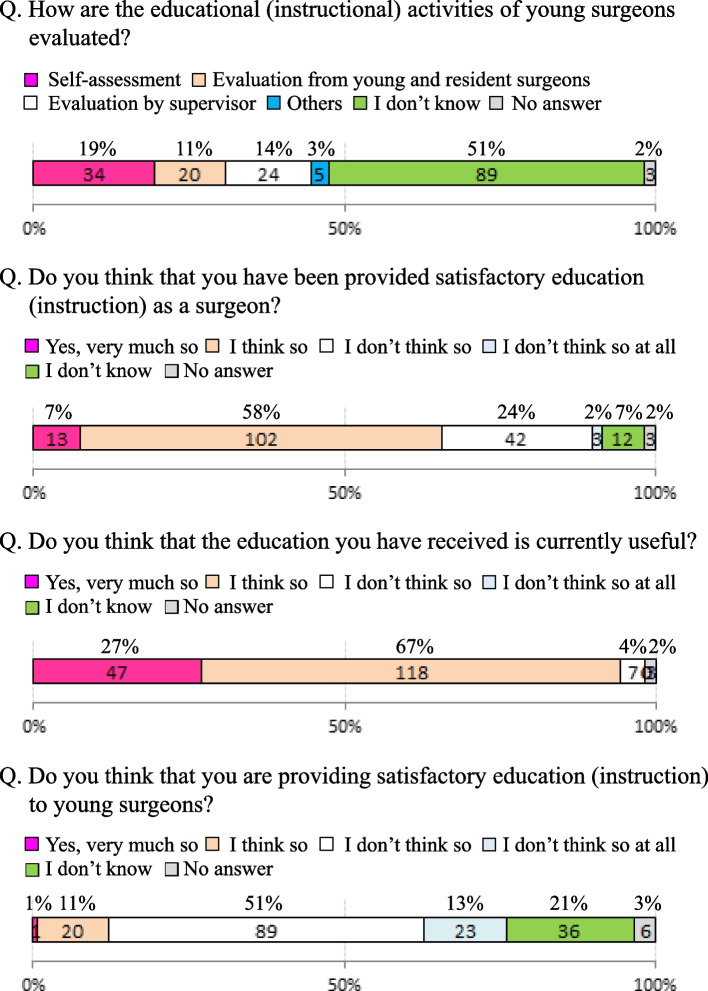
Assessment of the education/instruction of young surgeons

- (1)-4. Evaluation methods for educational training (Fig. 3)

Regarding the methods used to evaluate mentors who are educating young surgeons, although “self-assessment (19%)”, “evaluation by supervisor (14%)”, and “evaluation from young and resident surgeons (11%)” were cited, the most frequent answer was “I don’t know (51%)”.

When considering the surgical education that the surgeons themselves received, 65% of all respondents were satisfied and 26% were dissatisfied. Concerning the impact of the surgical education that they received, 94% felt it was quite useful. However, in terms of their satisfaction with the surgical education they themselves provided to young surgeons, “satisfaction” was indicated by 12% but “dissatisfaction” was indicated by 64%.

### Questionnaire survey (2) survey on the current status of surgical education for young surgeons

- (2)-1. Response rate

The total number of valid respondents was 87, giving a response rate of 93%; 27 (31%) were young surgeons under the age of 40 years and 60 (69%) were mentors of young surgeons.

- (2)-2. Background of respondents

The mean age of the respondents was 32 years for young surgeons and 52 years for mentors. There were 57 surgeons working in urban areas (20 young surgeons and 37 mentors) and 30 surgeons working in the region (7 young surgeons and 23 mentors).

**Fig. 4:**
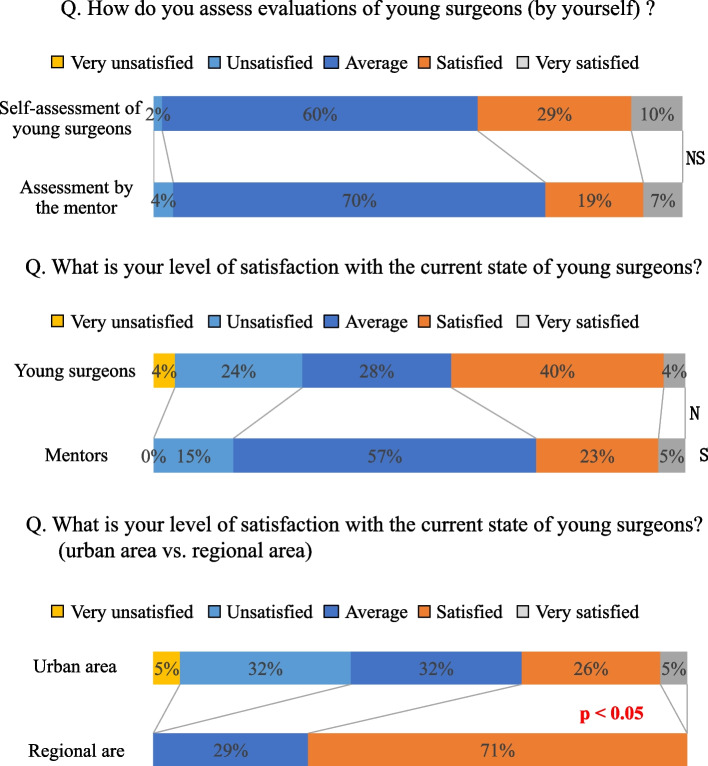
Level of satisfaction with the current state of young surgeons

- (2)-3. Regarding the level of satisfaction with the current status of surgical education for young surgeons (Fig. 4)

When considering the medical evaluation of young surgeons, 39% of the young surgeons were satisfied with self-evaluation, whereas 26% of the mentors were satisfied, which was not significantly different. Also, the level of satisfaction with the current status of surgical education for young surgeons (44% were satisfied) did not significantly differ with that of their mentors (28%). In contrast, when comparing young surgeons between those working in urban versus regional areas, 37% of young surgeons in urban areas were dissatisfied with their current status compared to 0% in the regional area (*p* < 0.05).

- (2)-4. Mutual expectations between young surgeons and their mentors

**Fig. 5:**
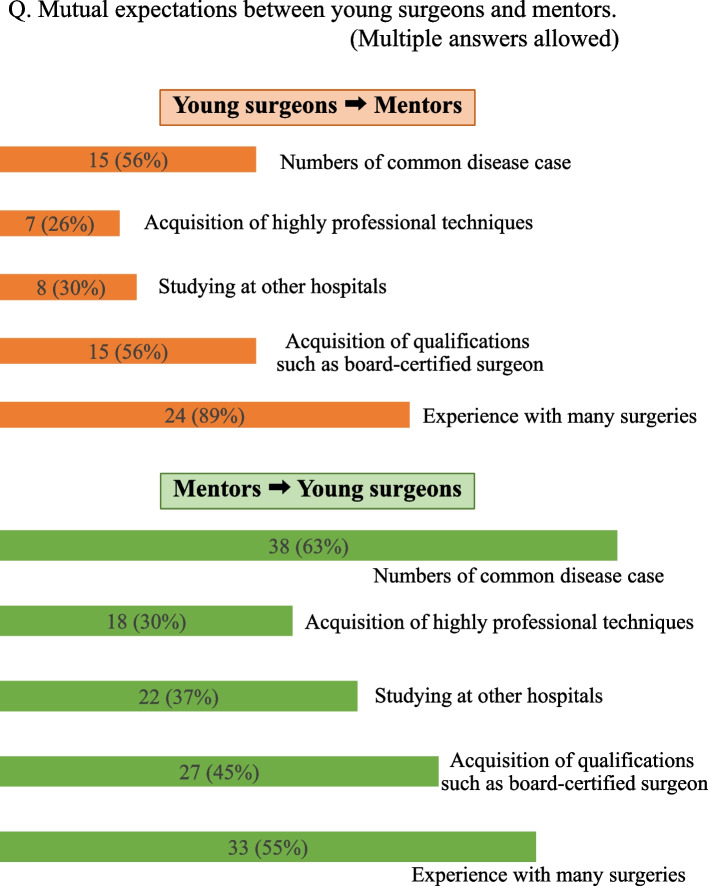
Mutual expectations between young surgeons and their mentors

Questions that allowed multiple answers were described as multiple answers. Regarding what young surgeons expect from their mentors (multiple answers), “experience with many surgeries (89%)” was the most common response, followed by “number of common disease case” and “acquisition of qualification such as board-certified surgeon” (both 56%). Conversely, the expectations of mentors for young surgeons were “number of common disease case (63%)”, “experience with many surgeries (55%)”, and “acquisition of qualifications such as board-certified surgeon (45%)” (Fig. 5). Also, 89% of young surgeons were willing to work in a regional area, and 72% of mentors likewise wanted them to do this.

## Discussion

We conducted our survey from two perspectives, one on the awareness regarding the education of young surgeons, the other on the current status of surgical education for young surgeons. Although the interval was seven years, we decided to report these results together so that both can be compared. The present questionnaire surveys on surgical education showed that approximately 90% of the responding surgeons had experience in providing surgical education and 75% of surgeons were interested in teaching. Over 60% of the surgeons were satisfied with the surgical education they received, but only 10% of the surgeons were satisfied with the education they provided to young surgeons. Furthermore, more than 60% of surgeons stated that there was no established method to evaluate their own surgical education. However, there was no difference in each group’s satisfaction and mutual evaluation between young surgeons and their mentors, as both of them expected the other to have experience with many cases and operations.

In recent years, the number of physicians in Japan has continued to increase. In contrast, although the number of surgeons had been increasing gradually since 2006, it has shown a downward trend since 2012. Even after the new specialty board system was begun, there is still an uneven distribution of surgeons in regional areas because surgeons are excluded from the new ceiling system, which sets an upper limit on the number of specialists, in Japan [3]. Moreover, the program standards of the new Board-Certified Surgeon system, which started in 2018, also determine the recruitment of physicians specializing in surgery according to the number of surgical cases and mentors, so it is assumed that a further concentration of surgeons in urban areas will continue in the future. However, the work-style reforms for physicians, which are to be introduced in 2024, may limit the working hours of surgeons. Young surgeons working in the region need to study in a small number of cases within a limited time. Thus, as the social circumstances surrounding surgeons are changing, surgeons working in the region must also provide education to young surgeons as well as maintain and improve the quality of their own surgical skills in daily clinical practice.

The most impressive result of this questionnaire survey was the low self-evaluation of the mentors’ own surgical education. To date, although there have been some reports on methods of evaluating surgical techniques, there are very few reports regarding the evaluation of the mentors’ own methods of surgical education. Gearhart et al. compared assessments of training methods for surgical procedures between program directors and graduates of training programs in pediatric urology and colorectal surgery [4]. However, no assessments of the program directors’ educational methods were mentioned. Sugiura et al. performed an observational assessment of clinical training for first-year postgraduate trainees and reported that both the proper training of mentors and a good relationship between mentors and trainees were necessary to make appropriate assessments [5]. But again, there was no mention of the assessment of the mentors themselves. It is clear that the trainee’s assessment of the mentor is useful in improving the educational system [6]. Gardner AK, et al. also reported that there is a reciprocity effect in the faculty and resident evaluation process in the surgical residency environment [7]. Currently, in the Online Clinical Education and Assessment System for Postgraduate Clinical Residents (E-POrtfolio of Clinical training [EPOC2]), which is the tool for the assessment of postgraduate clinical residents in Japan, mentors are also being evaluated by residents. It is also necessary to establish an evaluation system for mentors in surgical education, and it is believed that this system will enable mentors to engage in surgical education with confidence.

Although regional disparities in healthcare are often discussed, there are only a few reports about disparities between urban and regional areas in surgical education. Fonseca AL, et al. reported that lack of confidence of graduating general surgery residents in performing open vascular maneuvers varied regionally and was associated with both demographic and program-specific factors [8]. Kang MJ, et al. described that because there are the disparities in their regional distribution of the surgical specialists in Ghana, the current state of training at teaching hospitals should be investigated to decide where and how long the surgical rotation should take place [9]. A nationwide questionnaire survey on acquisition of Board-Certified Surgeon status in the Japan Surgical Society showed that surgeons in regional areas tend to have less experience with surgical operations and fewer opportunities to undergo regular off-the-job training outside the operating room than those in urban areas [10]. However, in our study, the level of satisfaction with surgical education was higher among young surgeons working in the regional area than in urban areas, and both mentors and young surgeons wanted to work in the region. Yoshimura et al. reported that there were large disparities among facilities regarding the training environment of pediatric cardiac surgeons, and those surgeons with a high number of surgical cases were more satisfied [11]. Our survey suggested that even if the numbers of surgical cases in a regional area are lower than those in urban areas, higher satisfaction of young surgeons can be achieved. Therefore, it was considered that young surgeons would be more satisfied if they could perform the operation even if the number of cases was small. We believe that providing safe and effective surgical education for young surgeons can also contribute to greatly improving their surgical techniques, which in turn can lead to improvement the shortage of qualified surgeons.

There are some limitations in this study. First, this study was conducted only in a single prefecture in Japan. The bias in the results couldn’t be removed. Second, this survey didn’t ask about the knowledge of specific educational methods, such as simulation and animal lab.

Based on this study, a nationwide survey including various levels of institutions in regions may facilitate the development of a better surgical education curriculum.

## Conclusion

In the present survey, although mentor surgeons earnestly carried out the education of young surgeons, it was not possible to carry it out with full confidence due to the lack of a system to evaluate the mentors’ educational methods. Development of an evaluation system for surgical education is necessary for young surgeons so that they can receive effective surgical education.

## Data Availability

The datasets analyzed during the current study are available from the corresponding author upon reasonable request.
